# Development and validation of an algorithm for identifying patients undergoing dialysis from patients with advanced chronic kidney disease

**DOI:** 10.1007/s10157-024-02614-3

**Published:** 2025-01-06

**Authors:** Takahiro Imaizumi, Takashi Yokota, Kouta Funakoshi, Kazushi Yasuda, Akiko Hattori, Akemi Morohashi, Tatsumi Kusakabe, Masumi Shojima, Sayoko Nagamine, Toshiaki Nakano, Yong Huang, Hiroshi Morinaga, Miki Ohta, Satomi Nagashima, Ryusuke Inoue, Naoki Nakamura, Hideki Ota, Tatsuya Maruyama, Hideo Gobara, Akira Endoh, Masahiko Ando, Yoshimune Shiratori, Shoichi Maruyama

**Affiliations:** 1 Department of Nephrology, Nagoya University Graduate School of Medicine, 65 Tsurumai–cho, Showa–ku, Nagoya, Aichi 464–8550 Japan; 2 Department of Advanced Medicine, Nagoya University Hospital, Nagoya, Japan; 3 Institute of Health Science Innovation for Medical Care, Hokkaido University Hospital, Sapporo, Japan; 4 Kyusyu University Hospital, Fukuoka, Japan; 5 Department of Medicine and Clinical Science, Graduate School of Medical Sciences, Kyushu University, Fukuoka, Japan; 6 Division of Medical Informatics, Okayama University Hospital, Okayama, Japan; 7 Department of Comprehensive Therapy for Chronic Kidney Disease, Faculty of Medicine, Dentistry and Pharmaceutical Sciences, Okayama University, Okayama, Japan; 8 Clinical Research Promotion Center, The University of Tokyo Hospital, Tokyo, Japan; 9 Department of Healthcare Information Management, The University of Tokyo Hospital, Tokyo, Japan; 10 Medical Information Technology Center, Tohoku University Hospital, Sendai, Japan; 11 Department of Medical Informatics, Hokkaido University Hospital, Sapporo, Japan; 12 Medical IT Center, Nagoya University Hospital, Nagoya, Japan

**Keywords:** Chronic kidney disease, Algorithm, Classification, Dialysis

## Abstract

**Background:**

Identifying patients on dialysis among those with an estimated glomerular filtration rate (eGFR) < 15 mL/min/1.73 m^2^ remains challenging. To facilitate clinical research in advanced chronic kidney disease (CKD) using electronic health records, we aimed to develop algorithms to identify dialysis patients using laboratory data obtained in routine practice.

**Methods:**

We collected clinical data of patients with an eGFR < 15 mL/min/1.73 m^2^ from six clinical research core hospitals across Japan: four hospitals for the derivation cohort and two for the validation cohort. The candidate factors for the classification models were identified using logistic regression with stepwise backward selection. To ensure transplant patients were not included in the non-dialysis population, we excluded individuals with the disease code Z94.0.

**Results:**

We collected data from 1142 patients, with 640 (56%) currently undergoing hemodialysis or peritoneal dialysis (PD), including 426 of 763 patients in the derivation cohort and 214 of 379 patients in the validation cohort. The prescription of PD solutions perfectly identified patients undergoing dialysis. After excluding patients prescribed PD solutions, seven laboratory parameters were included in the algorithm. The areas under the receiver operation characteristic curve were 0.95 and 0.98 and the positive and negative predictive values were 90.9% and 91.4% in the derivation cohort and 96.2% and 94.6% in the validation cohort, respectively. The calibrations were almost linear.

**Conclusions:**

We identified patients on dialysis among those with an eGFR < 15 ml/min/1.73 m^2^. This study paves the way for database research in nephrology, especially for patients with non-dialysis-dependent advanced CKD.

**Supplementary Information:**

The online version contains supplementary material available at 10.1007/s10157-024-02614-3.

## Introduction

In diseases such as chronic kidney disease (CKD), where it takes a long time to reach traditional endpoints [1, 2], observational studies are an important source of evidence [3–6]. The primary data sources for observational studies include cohorts [7–9], registries [10, 11], and data generated from routine clinical practice, also known as real-world data (RWD) [12, 13]. Classical randomized controlled trials (RCTs) typically involve the prospective collection of high-quality information from patients with specific attributes in a methodologically rigorous manner to ensure high internal validity. However, classical clinical trials often enroll a very limited population to assess the efficacy and safety of treatments despite enormous development costs. Given the diversity of patients with CKD who present with varying etiologies, disease progression, and complications [14, 15], there is a growing demand for evaluating the efficacy and safety of treatments tailored to individual patients, a concept also known as personalized medicine. Conducting RCTs to meet these diverse needs is impractical, and the complementary use of RWD is increasingly in demand [16, 17].

RWD sources derived from routine clinical practice in Japan are generally categorized into claims and hospital-based data such as electronic health records (EHRs) [18]. Unlike other RWD sources, clinical research using EHR data can incorporate laboratory results, which has led to significant advancements in the study of kidney diseases in recent years [19]. The Japan Chronic Kidney Disease Database (J-CKD-DB), established in 2014, produced a series of important research findings [20–22]. Notably, an observational study of sodium-glucose cotransporter-2 inhibitors demonstrated their efficacy in a real-world setting [23], making them a valuable precedent in Japan. However, the database currently lacks information on kidney replacement therapy (KRT), requiring the use of an eGFR < 15 mL/min/1.73 m^2^ as a surrogate endpoint. Consequently, patients with an eGFR < 15 mL/min/1.73 m^2^ could not be included. Several clinical trials on advanced CKD stages (G4 and G5) have been conducted in recent years [24–27], leading to a growing demand for evidence using RWD. Indeed, the eGFR at the time of initiating KRT in Japan and Taiwan is as low as 5–6 mL/min/1.73 m^2^, compared to approximately 10 mL/min/1.73 m^2^ in the United States and European countries [28, 29], suggesting that patients with CKD in these regions endure a prolonged G5 stage. However, the proportion of patients with an eGFR < 15 mL/min/1.73 m^2^ who are on dialysis remains unclear in real-world clinical settings.

To further facilitate the generation of real-world evidence (RWE) utilizing EHR databases in Japan, we aimed to investigate the dialysis status in patients with an eGFR < 15 mL/min/1.73 m^2^ and to develop algorithms for classifying whether a patient is currently undergoing dialysis, using data from the clinical research core hospitals across Japan. Such classification models would enhance RWE studies using EHR databases in Japan, particularly where dialysis profile information is not directly available.

## Materials and methods

### Data source and patient selection

Each institution has its own database or data warehouse. Data were collected from six clinical research core hospitals: Hokkaido University Hospital, Tohoku University Hospital, The University of Tokyo Hospital, Nagoya University Hospital, Okayama University Hospital, and Kyushu University Hospital. In these hospitals, most patients undergo in-center maintenance dialysis at neighboring hospitals or clinics. The clinical research core hospitals are designated medical institutions in Japan that play a central role in advancing high-quality clinical research and investigator-initiated trials at the international level. These hospitals are crucial for developing innovative pharmaceuticals, medical devices, and medical technologies originating from Japan.

First, patients who had an eGFR value of < 60 ml/min/1.73 m^2^ at any point during the window period (April 1, 2018, to September 30, 2018) were enrolled and measured at least three times until March 31, 2020, with 730 ± 90 days apart between the oldest and the newest dates. The index date was defined as the date of the first measurement of eGFR during the window period. The primary analysis aimed to develop and validate classification models using data from patients with an eGFR value < 15 mL/min/1.73 m^2^. To exclude patients who had undergone kidney transplantation, we removed those with the disease code Z94.0. We also investigated the records of all patients at Nagoya University Hospital to obtain information on whether they had undergone KRT and compared the baseline characteristics among patients with an eGFR > 60, 15–60, and < 15 mL/min/1.73 m^2^.

The first four hospitals were designated as the derivation cohorts and the latter two hospitals were designated as the validation cohorts. This study was conducted in accordance with the principles of the Declaration of Helsinki. The ethical review board approved the study protocol. The requirement of written informed consent was waived. The classification model was developed in accordance with the TRIPOD statement [30].

### Clinical data

Clinical data including laboratory parameters, prescription medicine, and disease name codes were collected 30 days before the index date. The laboratory parameters included complete blood counts including white and red blood cells (WBC and RBC), hemoglobin, hematocrit, mean corpuscular volume (MCV), mean corpuscular hemoglobin (MCH), mean corpuscular hemoglobin concentration (MCHC), and platelet count, total protein, serum albumin, transaminase, alkaline phosphatase (ALP), gamma-glutamyl transpeptidase (γ-GTP), lactate dehydrogenase (LDH), blood uric nitrogen (BUN), eGFR, uric acid (UA), sodium, potassium, phosphorus, calcium, magnesium, creatine kinase (CK), brain natriuretic peptide (BNP), C–reactive protein (CRP), cholinesterase, total, high- and low-density lipoprotein (LDL and HDL) cholesterol, triglyceride, and total bilirubin. ALP and LDH levels were measured using the Japan Society of Clinical Chemistry (JSCC) methods. We defined Na–Cl gap as the values of sodium minus chloride.

Prescription medicines included medicines that were prescribed only in that hospital and were classified based on the World Health Organization Anatomical Therapeutic Chemical (WHO–ATC) codes (Supplementary Table 1). PD solutions were identified using the Therapeutic Category of “3420.” Disease names were coded using the International Classification of Diseases 10th Revision (ICD-10) codes. We selected the kidney disease–related ICD-10 codes listed in Supplementary Table 2.

### Data acquisition of dialysis information through chart review

Local investigators identified patients who underwent any form of KRT through a medical chart review. Patients whose baseline eGFR value was < 15 mL/min/1.73 m^2^ were designated for chart review. Medical chart reviews were performed by investigators with a medical doctor’s license. KRT includes hemodialysis (HD), peritoneal dialysis (PD), and kidney transplantation. PD combined with HD was classified as PD. The outcome of interest was a prevalent HD or PD at the index date. Prevalent dialysis refers to ongoing maintenance dialysis treatment provided in a dialysis facility or at home and does not include dialysis for temporary kidney impairment, such as in acute kidney injury.

### Development of algorithms to classify patients undergoing dialysis treatment at baseline

To explore candidates for classification models, we performed logistic regression analysis to examine the association between factors and the outcome, adjusted for age, sex, and eGFR at baseline. We further explored the factors associated with the outcome using a stepwise approach with backward selections: removing factors with *P*-values > 0.15. Because the assignment of disease codes at each facility was not standardized and given that prescription medications were only prescribed at that facility, we could not ascertain prescription information at other facilities. Therefore, we utilized laboratory data to develop the classification models for the primary analysis. We then added disease names and prescription information as candidates for the development of the classification models.

### Statistical analysis

Baseline characteristics were summarized by stratifying the patients who were undergoing dialysis treatment at baseline for both derivation and validation cohorts. Continuous variables are presented as mean (standard deviations) or median (interquartile ranges) and categorical variables are reported as numbers (%). Intergroup comparisons were conducted using the chi-squared test for categorical variables and either the Student’s *t*-test or Wilcoxon rank-sum test for continuous variables. Logistic regression analysis was used to examine the association of laboratory parameters, prescription medicine, and disease name codes with the outcome. We used variables that were available to > 80% of the participants. Among the factors strongly correlated with each other, we selected the variables with the lowest P-values. Candidate factors for the classification models were obtained using stepwise backward selection. Missing values were addressed through multiple imputations with chained equations, resulting in 100 datasets for estimating prediction scores. The discrimination performance was assessed using the area under receiver operating characteristic curves (AUCs) and the cutoff values were calculated using the Youden index. The primary model was selected based on the highest AUC observed in the derivation cohort. Calibration plots were used to compare between observed frequency and the predicted probabilities. To assess the relative impact of each parameter, the values were standardized by dividing them by the standard deviation and the standardized estimates were calculated using logistic models. Statistical significance was set at *P* < 0.05. All statistical analyses were performed using Stata MP 18.0 (StataCorp., TX, USA).

## Results

### Patient selection and baseline characteristics

**Fig. 1:**
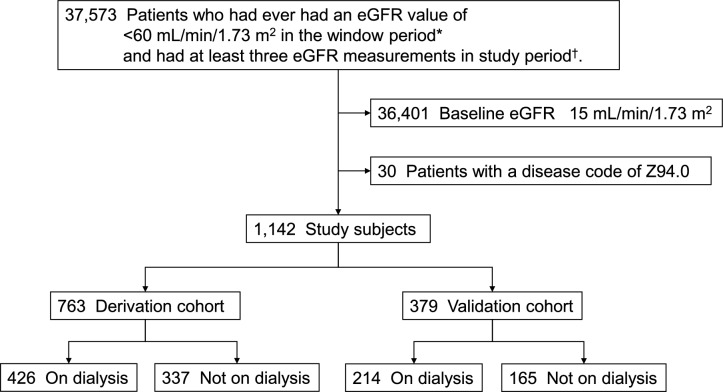
Patient flow diagram**.** The number of patients who had ever had an eGFR value of < 60 mL/min/1.73 m^2^ was 37,573. After exclusion, 1142 eligible patients were divided into the derivation cohort (*n* = 763) and the validation cohort (*n* = 379). *Window period: Between April 1, 2018, and September 30, 2018. ^†^Study period: Between April 1, 2018, and March 31, 2020

We obtained data from 37,573 patients from six hospitals. Of the 37,573 patients, the number of patients with an eGFR > 60, 15–60, and < 15 mL/min/1.73 m^2^ was 4891, 31,510, and 1172, respectively. Among the 1172 patients with an eGFR < 15 mL/min/1.73 m^2^, we further excluded those with the code Z94.0 (*n* = 30). This resulted in 1,142 patients, of whom 640 patients (56%) were currently undergoing HD or PD (Fig. 1).

**Fig. 2:**
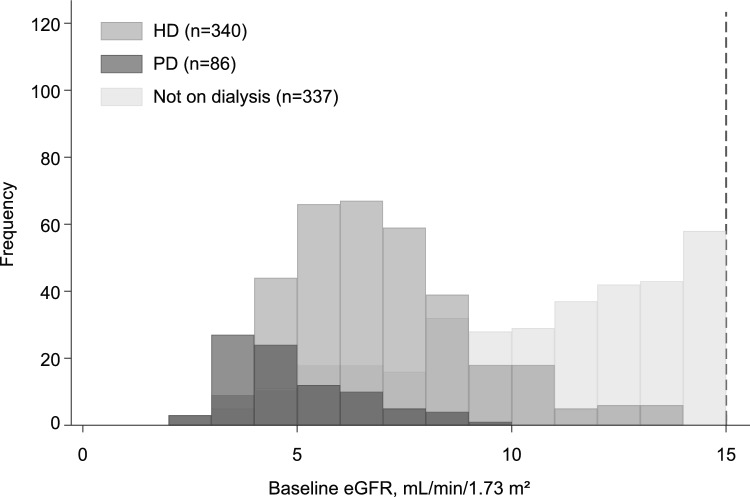
Distribution of estimated glomerular filtration rate across different modalities of kidney replacement therapy**.** The median values of estimated glomerular filtration rate were 6.5, 4.6, and 11.2 mL/min/1.73 m^2^ for patients on HD, PD, and not on dialysis, respectively

We included 763 patients (426 on dialysis; 55.8%) in the derivation cohort and 379 patients (214 on dialysis; 56.5%) in the validation cohort. The baseline characteristics of the patients are shown in Table 1. The distribution of eGFR across the different modalities of KRT is shown in Fig. 2. Patients undergoing PD had the lowest eGFR, followed by those on HD. Patients on dialysis were more likely to have higher values of hemoglobin, BUN, and Na–Cl gap, and lower values of UA. Regarding the disease name code, N18.0, which was no longer valid during the study period, was dominant in patients on dialysis in the derivation cohort, but not in the validation cohort. Patients on dialysis were more likely to have the code of T85.7 in both cohorts. Regarding medication information, PD solutions were only prescribed for patients on dialysis, whereas antihypertensive medications were less likely to be prescribed to patients on dialysis.

**Table 1:** Baseline characteristics of the derivation and validation cohorts

	Derivation cohort					Validation cohort				
	*N*	Total (*N* = 763)	HD or PD (*N* = 426)	Not on dialysis (N = 337)	*P* value	*N*	Total (*N* = 379)	HD or PD (*N* = 214)	Not on dialysis (*N* = 165)	*P* value
Age, years	763	64.1 (13.1)	63.3 (12.8)	65.2 (13.3)	0.042	379	61.0 (13.7)	60.7 (13.2)	61.4 (14.5)	0.60
Male sex	763	481 (63)	288 (68)	193 (57)	0.003	379	214 (56)	123 (57)	91 (55)	0.65
White blood cell, × 10^6^/μl	726	6.35 (2.39)	6.24 (2.39)	6.48 (2.39)	0.19	379	6.35 (2.63)	6.27 (2.66)	6.46 (2.61)	0.48
Red blood cell, × 10^6^/μl	726	3.79 (0.60)	3.86 (0.61)	3.69 (0.57)	< 0.001	379	3.67 (0.53)	3.79 (0.55)	3.52 (0.47)	< 0.001
Hemoglobin, g/dL	726	11.4 (1.6)	11.7 (1.5)	11.0 (1.6)	< 0.001	379	11.1 (1.5)	11.5 (1.5)	10.7 (1.3)	< 0.001
Hematocrit, %	726	35 (5)	36 (5)	34 (5)	< 0.001	379	35 (5)	36 (5)	33 (4)	< 0.001
MCH, pg	720	30 (2)	31 (3)	30 (2)	0.005	379	30 (2)	30 (3)	30 (2)	0.90
MCHC, %	718	32 (1)	32 (1)	32 (1)	0.016	379	32 (1)	32 (1)	32 (1)	0.042
MCV, fL	721	94 (7)	95 (7)	93 (6)	< 0.001	379	95 (7)	95 (8)	94 (6)	0.40
Platelet, × 1000/μl	726	203 (100)	208 (115)	198 (78)	0.18	379	210 (81)	200 (76)	223 (85)	0.006
Total Protein, mg/dL	469	6.8 (0.7)	6.7 (0.7)	6.9 (0.6)	0.054	371	6.8 (0.7)	6.7 (0.7)	6.8 (0.7)	0.29
Albumin, mg/dL	701	3.7 (0.5)	3.6 (0.5)	3.8 (0.5)	< 0.001	376	3.8 (0.5)	3.7 (0.5)	3.8 (0.6)	0.12
HbA1c, %	317	6.0 (5.5–6.7)	5.9 (5.3–6.8)	6.0 (5.6–6.6)	0.29	203	5.8 (5.3–6.6)	5.7 (5.2–6.6)	5.9 (5.4–6.7)	0.19
ALT, U/L	702	15 (54)	13 (11)	19 (81)	0.19	376	16 (23)	14 (11)	17 (32)	0.19
AST, U/L	676	19 (55)	16 (10)	22 (82)	0.13	378	19 (19)	18 (13)	21 (25)	0.20
ALP (JSCC), U/L	621	240 (189–321)	253 (192–334)	233 (180–311)	0.021	363	255 (192–356)	260 (190–367)	247 (194–333)	0.50
γ-GTP, U/L	653	20 (14–35)	20 (13–36)	20 (14–33)	0.92	369	21 (14–41)	22 (14–42)	20 (14–36)	0.30
LDH (JSCC), U/L	624	198 (169–232)	197 (167–228)	199 (169–234)	0.40	368	200 (170–238)	201 (168–238)	199 (172–238)	0.57
BUN, mg/dL	724	49.5 (18.2)	43.5 (16.5)	57.0 (17.4)	< 0.001	379	52.3 (20.9)	45.5 (18.4)	61.2 (20.5)	< 0.001
eGFR, mL/min/1.73 m^2^	763	7.5 (5.6–11.0)	6.0 (4.9–7.6)	11.1 (8.3–13.1)	< 0.001	379	7.0 (5.0–10.0)	6.0 (4.7–7.0)	10.0 (8.0–13.0)	< 0.001
Uric acid, mg/dL	657	5.8 (4.6–6.8)	5.0 (4.0–6.2)	6.3 (5.3–7.4)	< 0.001	348	5.9 (4.7–7.0)	5.4 (4.2–6.3)	6.4 (5.6–7.5)	< 0.001
Sodium, mEq/L	678	140 (138–141)	139 (137–141)	140 (138–142)	0.002	372	139 (137–141)	139 (137–141)	140 (137–141)	0.045
Chrolide, mEq/L	645	103 (100–106)	101 (98–103)	106 (103–108)	< 0.001	370	104 (100–107)	101 (99–104)	107 (104–109)	< 0.001
Na – Cl, mEq/L	645	37 (34–39)	39 (37–41)	34 (32–36)	< 0.001	370	36 (33–38)	38 (36–39)	33 (31–35)	< 0.001
Potassium, mEq/L	721	4.7 (0.7)	4.7 (0.7)	4.8 (0.6)	0.015	372	4.6 (0.7)	4.5 (0.7)	4.8 (0.6)	< 0.001
Ca, mg/dL	628	8.8 (8.4–9.2)	8.9 (8.4–9.3)	8.8 (8.4–9.2)	0.19	352	8.8 (8.4–9.2)	9.0 (8.6–9.4)	8.7 (8.3–9.0)	< 0.001
Phosphorus, mg/dL	540	4.4 (3.9–5.1)	4.7 (4.0–5.5)	4.3 (3.8–4.7)	< 0.001	301	4.5 (3.8–5.2)	4.6 (3.8–5.4)	4.5 (3.8–5.1)	0.25
Magnesium, mg/dL	304	2.1 (1.9–2.3)	2.2 (2.0–2.3)	2.0 (1.8–2.2)	< 0.001	173	2.1 (1.9–2.4)	2.3 (2.0–2.5)	2.0 (1.8–2.2)	< 0.001
CK, U/L	475	92 (58–147)	74 (51–121)	114 (79–196)	< 0.001	276	95 (60–150)	72 (54–126)	113 (78–188)	< 0.001
BNP, pg/ml	259	97 (46–243)	93 (49–340)	112 (41–193)	0.25	79	113 (51–379)	137 (70–530)	83 (43–162)	0.022
CRP, mg/dL	606	0.11 (0.04–0.41)	0.13 (0.04–0.46)	0.09 (0.03–0.35)	0.040	224	0.16 (0.05–0.54)	0.19 (0.06–0.62)	0.10 (0.02–0.44)	0.043
Cholinesterase, U/L	270	246 (203–288)	238 (205–288)	253 (202–288)	0.55	246	246 (203–305)	250 (201–297)	243 (203–314)	0.79
Total cholesterol, mg/dL	445	171 (145–197)	171 (143–198)	171 (148–196)	0.59	313	175 (150–204)	173 (150–207)	176 (151–204)	0.97
HDL cholesterol, mg/dL	413	50 (41–64)	52 (42–64)	49 (40–64)	0.37	248	50 (38–59)	48 (38–59)	50 (41–59)	0.27
LDL cholesterol, mg/dL	376	90 (71–116)	94 (70–120)	88 (72–109)	0.26	266	95 (73–117)	96 (71–120)	95 (75–115)	0.91
Triglyceride, mg/dL	475	114 (83–167)	108 (76–155)	123 (94–176)	0.003	304	122 (89–174)	121 (85–170)	125 (94–179)	0.38
Total bilirubin, mg/dL	543	0.4 (0.3–0.6)	0.4 (0.3–0.6)	0.5 (0.4–0.6)	0.20	356	0.4 (0.3–0.5)	0.4 (0.3–0.5)	0.5 (0.4–0.6)	0.032
Disease code (ICD-10 code)										
N18.0	763	272 (36)	216 (51)	56 (17)	< 0.001	379	1 (0)	1 (0)	0 (0)	0.38
N18.5	763	62 (8)	33 (8)	29 (9)	0.67	379	165 (44)	125 (58)	40 (24)	< 0.001
T85.7	763	73 (10)	69 (16)	4 (1)	< 0.001	379	12 (3)	12 (6)	0 (0)	0.002
Prescription medicine										
RAS inhibitors	763	215 (28)	67 (16)	148 (44)	< 0.001	379	122 (32)	52 (24)	70 (42)	< 0.001
α blockers	763	75 (10)	28 (7)	47 (14)	< 0.001	379	28 (7)	13 (6)	15 (9)	0.27
β blockers	763	112 (15)	50 (12)	62 (18)	0.010	379	73 (19)	43 (20)	30 (18)	0.64
CCB	763	232 (30)	72 (17)	160 (47)	< 0.001	379	143 (38)	61 (29)	82 (50)	< 0.001
Diuretics	763	170 (22)	71 (17)	99 (29)	< 0.001	379	71 (19)	39 (18)	32 (19)	0.77
MRB	763	70 (9)	47 (11)	23 (7)	0.046	379	15 (4)	7 (3)	8 (5)	0.43
Anti-diabetics	763	150 (20)	76 (18)	74 (22)	0.16	379	66 (17)	35 (16)	31 (19)	0.54
NSAIDs	763	17 (2)	9 (2)	8 (2)	0.81	379	18 (5)	13 (6)	5 (3)	0.17
Potassium binders	763	81 (11)	12 (3)	69 (20)	< 0.001	379	50 (13)	15 (7)	35 (21)	< 0.001
Peritoneal dialysis solutions	763	36 (5)	36 (8)	0 (0)	< 0.001	379	52 (14)	52 (24)	0 (0)	< 0.001
Clinical setting	763				0.17					0.78
Outpatient		665 (87)	61 (86)	37 (89)			331 (87)	186 (87)	145 (88)	
Hospitalization		98 (13)	61 (14)	37 (11)		379	48 (13)	28 (13)	20 (12)	

Data are expressed as the mean (SD) or median (IQR) for continuous variables and n (%) for categorical variables. Statistical significance was set at *P* < 0.05

*WBC* White blood cell; *RBC* Red blood cell; *MCH* Mean corpuscular hemoglobin; *MCHC* Mean corpuscular hemoglobin concentration; *MCV* Mean corpuscular volume; *TP* Total protein; *AST* Aspartate aminotransferase; *ALT* Alanine aminotransferase; *ALP* Alkaline phosphatase; *JSCC* Japan Society of Clinical Chemistry; *γ-GTP* Gamma-glutamyl transpeptidase; LDH Lactate dehydrogenase; *BUN* Blood urea nitrogen; *eGFR* Estimated glomerular filtration rate; *Mg* Magnesium; *CK* Creatine kinase; *BNP* Brain natriuretic peptide; *CRP* C-reactive protein; *HDL* High-density lipoprotein; *LDL* Low-density lipoprotein; *RAS* Renin-angiotensin system; *CCB* Calcium-channel blockers; *MRB* Mineralocorticoid receptor blockers; *NSAIDs* Non-steroidal anti-inflammatory drugs

We also examined the patient data from Nagoya University Hospital. Of the 7435 patients, the number of patients with an eGFR > 60, 15–60, and < 15 mL/min/1.73 m^2^ was 1251, 5965, and 217, respectively. Most of the patients on HD or PD were included in the eGFR < 15 mL/min/1.73 m^2^ group (114/117 [97.4%]), whereas all patients who had undergone kidney transplantation were in the eGFR ≥ 15 mL/min/1.73 m^2^ group. Patients in the eGFR < 15 mL/min/1.73 m^2^ group were low in hemoglobin, albumin, cholinesterase, and total, HDL, and LDL cholesterol, and high in BUN, UA, phosphorus, BNP, and CRP (Supplementary Table 3).

### Developing a classification model

Although PD solutions were not prescribed to all patients on PD, all patients for whom PD solutions were prescribed were on HD or PD, that is, PD solutions perfectly classified patients on dialysis. After excluding patients prescribed PD solutions, factors associated with dialysis are shown in Supplementary Table 4. Multivariable-adjusted logistic regression analyses led to the classification model including following factors (Table 2): Model 1 included MCH, MCHC, albumin, BUN, eGFR, Na–Cl gap, and UA; Model 2 included RBC, hematocrit, albumin, BUN, eGFR, Na–Cl gap, and UA after excluding MCH and MCHC; and Model 3 included albumin, BUN, eGFR, Na–Cl gap, and UA. In Model 1, the odds ratios per standard deviation (95% confidence intervals) were substantially high for the Na–Cl gap (2.54 [1.83–3.52]) and low for eGFR (0.16 [0.12–0.23]) and BUN (0.27 [0.20–0.38]).

**Table 2:** Regression coefficients and standardized odds ratios in each Model in the derivation cohort

	Model 1		Model 2		Model 3		Model 4	
	AUC: 0.9508 (95% CI: 0.9341–0.9675)		AUC: 0.9487 (95% CI: 0.9315–0.9658)		AUC: 0.9500 (95% CI: 0.9331–0.9669)		AUC: 0.9593 (95% CI: 0.9449–0.9737)	
	Cut off point: 0.13621932		Cut-off point: 0.21675129		Cut off point: 0.40055719		Cut off point: 0.21814708	
	Regression coefficient	OR per SD	Regression coefficient	OR per SD	Regression coefficient	OR per SD	Regression coefficient	OR per SD
Albumin	− 0.8420557	0.66 (0.51–0.85)	− 0.8169266	0.67 (0.52–0.86)	− 0.9358818	0.63 (0.48–0.82)	− 0.8296468	0.66 (0.50–0.88)
BUN	− 0.0711404	0.27 (0.20–0.38)	− 0.0717403	0.27 (0.20–0.38)	− 0.0701483	0.28 (0.20–0.39)	− 0.0752203	0.26 (0.18–0.37)
eGFR	− 0.5460093	0.16 (0.12–0.23)	− 0.5441751	0.16 (0.12–0.23)	− 0.5433849	0.16 (0.12–0.23)	− 0.5031476	0.19 (0.13–0.27)
Na-Cl gap	0.2331772	2.54 (1.83–3.52)	0.2292866	2.50 (1.81–3.47)	0.2191977	2.40 (1.72–3.37)	0.1839276	2.09 (1.45–2.99)
Uric acid	− 0.267085	0.63 (0.47–0.84)	− 0.3107151	0.58 (0.44–0.78)	− 0.287648	0.61 (0.45–0.81)	− 0.2339113	0.66 (0.49–0.90)
MCH	− 0.3497718	1.51 (1.12–2.02)					0.1688315	1.50 (1.10–2.04)
MCHC	0.1703894	0.67 (0.49–0.90)					− 0.3714643	0.65 (0.47–0.89)
RBC					− 1.098978	0.52 (0.30–0.89)		
Hematocrit, %					0.1467186	2.08 (1.17–3.69)		
T85.7							1.357285	3.89 (1.04–14.6)
N18.0							1.467646	4.34 (2.38–7.94)
CCB							− 0.7974775	0.45 (0.23–0.88)
Constant	10.50713	–	4.68405	-	4.255136	-	12.27361	-

Prediction scores can be calculated using the regression coefficients for each model. ORs per SD were determined by dividing numerical variables by their respective SDs. *MCH* Mean corpuscular hemoglobin; *MCHC* Mean corpuscular hemoglobin concentration; *BUN* Blood urea nitrogen; *eGFR* Estimated glomerular filtration rate; *Na–Cl gap*, sodium-chloride gap; *AUC* Area under receiver operating characteristic curve; *OR* Odds ratio; *SD* standard deviation

We also added disease codes and prescription information into the model. In addition to the above-mentioned factors, disease codes T85.7 and N18.0, and calcium-channel blockers were selected (Model 4). The predicted probabilities of dialysis status and classification were calculated using the Supplementary calculator.

### Performance of the classification model

**Fig. 3:**
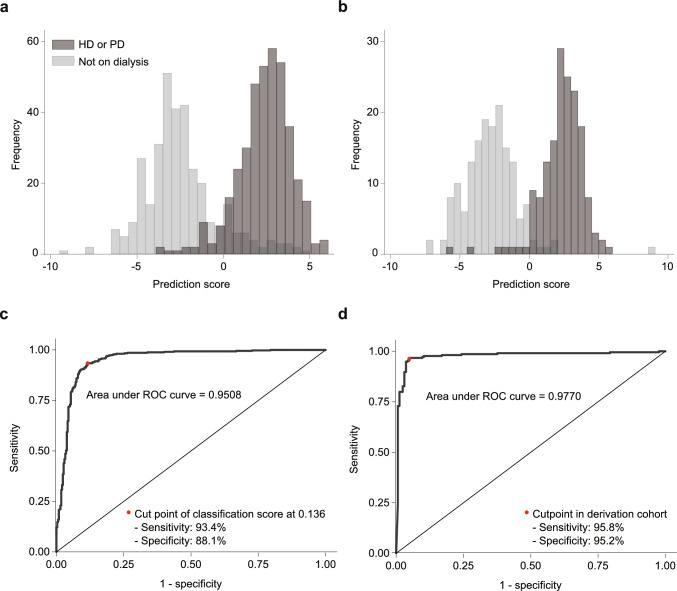
Distribution of prediction scores and receiver operating characteristic curves in the derivation and validation cohorts. Distribution of prediction scores in the derivation and validation cohorts (**a** and** b**, respectively). The scores were calculated using the following equation: Prediction score = 0.1383124 × MCH – 0.3254763 × MCHC – 0.7117655 × Alb – 0.0699673 × BUN – 0.5168784 × eGFR + 0.194965 × Na–Cl gap – 0.2364415 × UA + 10.90525. The receiver operating characteristic curves show excellent discrimination performance of the model at the cut-off point of – 0.147 in both the derivation and validation cohorts (**c** and **d**, respectively)

**Fig. 4:**
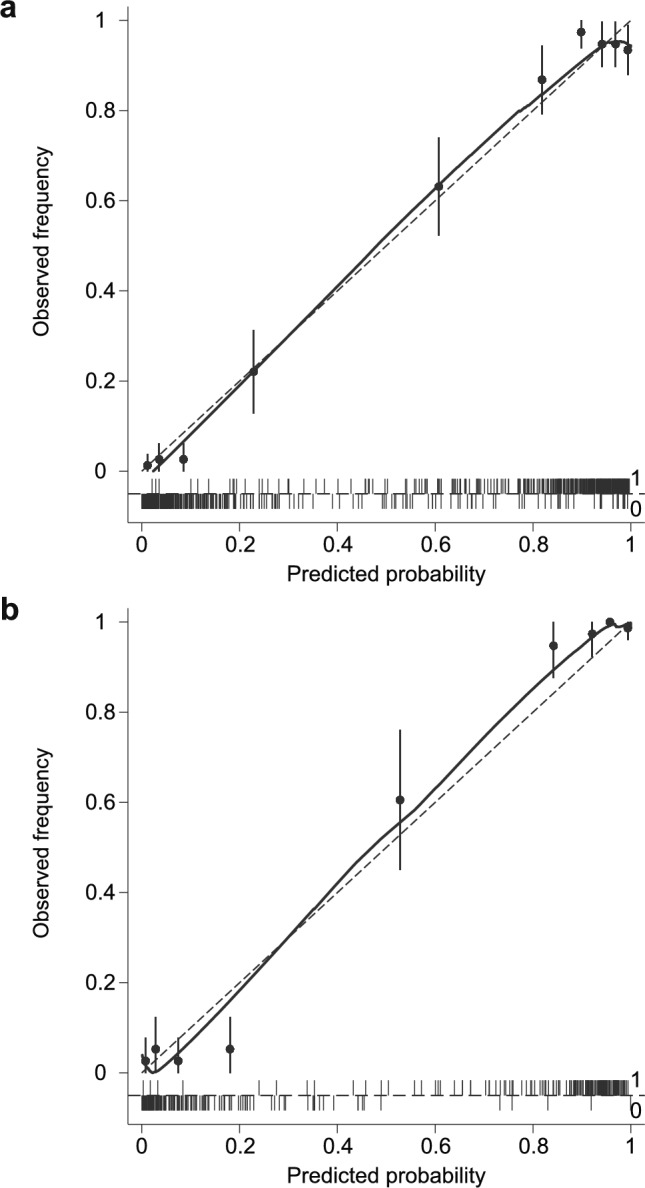
Calibration plots for observed frequency and predicted probability. **a** Derivation cohort. **b** Validation cohort

The prediction scores were distributed bimodally in both cohorts, and the AUCs were 0.95 and 0.98, indicating excellent discrimination (Fig. 3). The calibration plots showed an almost linear pattern, indicating excellent calibration (Fig. 4). The sensitivity, specificity, positive predictive value (PPV), and negative predictive value (NPV) were excellent in both cohorts, with values of 93.4%, 88.1%, 90.9%, and 91.4% in the derivation cohort and 95.8%, 95.2%, 96.2%, and 94.6% in the validation cohort, respectively) (Table 3). At each facility, the PPV was greater than 80% (Supplementary Table 5). The other models demonstrated comparable performance (Supplementary Table 6), and excluding hospitalized patients yielded similar results (Supplementary Table 7). When disease codes and prescription information were added to the model, the performance of the latter was slightly better; however, the difference in model performance was not substantial (Supplementary Fig. 1). Calibration plots also showed an almost linear pattern (Supplementary Fig. 2).

**Table 3:** Model performance

	HD or PD	Not on dialysis	Sensitivity	Specificity	PPV	NPV
Derivation cohort: AUC = 0.9508 (95% confidence interval: 0.9341–0.9675)						
≥ Cut point	398	40	93.4%	88.1%	90.9%	91.4%
< Cut point	28	297				
Validation cohort: AUC = 0.9770 (95% confidence interval: 0.9603–0. 9937)						
≥ Cut point	205	8	95.8%	95.2%	96.2%	94.6%
< Cut point	9	157				

*HD* Hemodialysis; *PD* Peritoneal dialysis; *PPV* Positive predictive value; *NPV* Negative predictive value; *AUC* Area under receiver operating characteristic curve

## Discussion

In this study, we clearly classified patients with an eGFR < 15 mL/min/1.73 m^2^ into those on dialysis and those not on dialysis with an excellent discrimination performance. This was meaningful in that this study paves the way for RWD analysis of non-dialysis-dependent advanced CKD including stage G5 in Japan. The PPVs were greater than 80% for all hospitals in this study, indicating that the models were highly versatile. To the best of our knowledge, this was the first chart review to use large-scale data from patients with CKD stage G5. The study findings are valuable in countries where the linkage between hospital-based data and public data reflecting dialysis status is challenging.

Kidney-related parameters (e.g., eGFR, BUN, and UA), anemia-related parameters (e.g., MCHC and MCH), and serum albumin were included in the classification models. The levels of eGFR were lower in patients undergoing dialysis than in those with non-dialysis-dependent CKD. This was reasonable because the levels of eGFR in patients who initiated dialysis were around 5 or 6 mL/min/1.73 m^2^ on average. BUN levels may be associated with uremia, which is particularly evident in late-stage non-dialysis-dependent CKD. The fact that the eGFR levels were low in patients on PD was also reasonable because patients on HD often visit tertiary-care educational hospitals the day after a dialysis session; thus, the levels of eGFR were higher in patients on HD than in those on PD. MCH and MCHC were included in the model because of the iron status and renal anemia in late-stage non-dialysis-dependent CKD patients. Anemia is often managed well with hematopoietic agents in patients on dialysis rather than in patients with non-dialysis-dependent CKD. Thus, variables related to anemia strongly contributed to this classification model. Hemoglobin levels and red blood cell counts were also associated with dialysis status; however, these variables were removed during the variable selection process because of their strong correlation.

The Na–Cl gap can be a surrogate of serum bicarbonate concentration [31], and a low Na–Cl gap indicates hyperchloremic metabolic acidosis without an anion gap, which is associated with the prognosis of patients with non-dialysis-dependent CKD [32]. In contrast, a high Na–Cl gap indicates a high level of serum bicarbonate, that is, metabolic alkalosis, or a counteraction of nonvolatile acid accumulation, such as ketone bodies, lactic acid, and uremic toxins, which is also known as metabolic acidosis with anion gap elevation [33]. Regarding metabolic alkalosis, post-dialysis-measured plasma bicarbonate is usually 2–5 units higher than the pre-dialysis concentration [34]. This can be affected by the prescribed dialysate bicarbonate concentration [35], and online HDF, which provides a large amount of bicarbonate, leading to metabolic alkalosis [36]. The type of dialysate and dialysis modality can affect plasma bicarbonate concentrations, even several hours after HD sessions [37]. Currently, nearly half of patients with HD undergo online HDF in Japan, suggesting that a certain percentage of patients may have metabolic alkalosis. The plasma bicarbonate concentrations were unavailable in this study; therefore, we plan to conduct a future study to determine whether these patients have truly metabolic alkalosis.

Incorporating disease codes and prescription information into the classification models slightly improved the classification performance. However, as the code of N18.0 was abolished during the study period, we may need to investigate the model performance using other disease codes in the future. Moreover, the code T85.7 may include dialysis catheter-related infections or complications, suggesting that we merely identified patients who had developed the complications. Regarding the prescription information, the prescription of PD solutions is straightforward, as it may represent patients currently on PD. The decreasing proportion of antihypertensive agent prescription in patients undergoing dialysis should be interpreted with caution. This may have included patients who were not undergoing dialysis at the university hospitals; therefore, this may not be useful in the future, when linkage to prescriptions at other hospitals becomes possible.

This study has several limitations. First, determining whether a patient was undergoing KRT was challenging if it was not recorded in the EHR. We requested that local investigators thoroughly review the medical records to capture this information as accurately as possible. Second, it was challenging to identify kidney transplant patients. If patients had a history of transplantation and regularly visited the hospital, their disease codes might have been registered; however, if they had not been registered, this information could have been missing. Even after excluding patients with the code Z94.0, there may still be kidney transplant patients in the non-dialysis population. Nevertheless, the number of patients with a history of transplantation was limited in the current dataset, resulting in only a minor impact on model performance. Third, the use of prescription medications and the assignment of disease codes could vary substantially across hospitals, and updates may be required owing to potential changes in the ICD-10 codes. Despite these variations, the performance of the model was consistent across different facilities when disease codes and prescription information were included. Fourth, although laboratory data are generally objective, the timing of blood sampling is not always consistent, which can affect the model accuracy. Prospective research is needed to collect clinical information with a defined schedule. Fifth, in this study, HD and PD were grouped together as ‘dialysis’ rather than classified separately. This approach was necessary because some patients currently undergoing HD had a long history of PD and continued to receive abdominal lavage with PD solutions to prevent encapsulating peritoneal sclerosis, and others were undergoing combined PD and HD. Due to the complexity of categorizing these cases, all patients on HD or PD were classified as being on dialysis. Sixth, restricting the study population to patients with at least three eGFR measurements during approximately 2 years of follow-up provided us with sufficient data to investigate hemodialysis status at the index date in stable CKD patients. We could not completely exclude the possibility of immortal-time bias by restricting the study population to survivors. Although our inclusion criteria helped increase the data reliability, we need to carefully consider selection bias and limited generalizability. Finally, the generalizability of this study may be limited by its focus on patients treated at clinical research core hospitals, which are tertiary teaching hospitals in Japan. Nevertheless, this study offers valuable insights, as it involved chart reviews of over 1000 patients with stage G5 CKD to evaluate dialysis profiles and develop classification models. Despite these challenges, the classification performance remained excellent.

## Conclusion

In this study, we developed and validated an algorithm that was capable of identifying patients undergoing dialysis using laboratory data extracted from EHRs. Our findings demonstrate that the proposed algorithm can accurately classify patients undergoing dialysis in those with CKD Stage G5. The high accuracy of this classification suggests that the algorithm can serve as a robust tool for future database studies that target populations with advanced CKD. By enabling more precise identification of these patients, this algorithm is expected to facilitate more extensive and rigorous research in this area, ultimately contributing to a deeper understanding and improved management of advanced CKD. Thus, this study provides a foundational framework upon which future research can build, marking an important step forward in the utilization of RWD in nephrology research.

## Supplementary Information

Below is the link to the electronic supplementary material.

Supplementary file1 (XLSX 467 KB)

Supplementary file2 (DOCX 421 KB)
