# Supplementary material for: Development and validation of an algorithm for identifying patients undergoing dialysis from patients with advanced chronic kidney disease
Source: Clin Exp Nephrol. 2025 Jan 6;29(5):650–61. doi: 10.1007/s10157-024-02614-3 (PMC12049401; doi:10.1007/s10157-024-02614-3)
Supplement: Supplementary file 2 — Supplementary file2 (DOCX 421 KB) [file 10157_2024_2614_MOESM2_ESM.docx]

**Development and Validation of Algorism for Database Research to Classify Patients undergoing Dialysis: Analysis from Rinchu net**

**Supplementary Material**

**Supplementary Tables**

[**Supplementary Table 1.** Medicine list](#_Toc182260533)

[**Supplementary Table 2.** Disease names and their International Classification of Diseases 10th Revision (ICD–10) codes](#_Toc182260534)

[**Supplementary Table 3.** Baseline characteristics and modality of kidney replacement therapy](#_Toc182260535)

[**Supplementary Table 4.** Logistic regression analysis to develop a classification model](#_Toc182260536)

[**Supplementary Table 5**. The model performance at each hospital](#_Toc182260537)

[**Supplementary Table 6.** Performance of other Models](#_Toc182260538)

[**Supplementary Table 7.** Performance of classification models after excluding hospitalized patients](#_Toc182260539)

**Supplementary Figures**

[**Supplementary Fig. 1** Distribution of prediction scores 4 and receiver operating characteristic curves in the derivation and validation cohorts](#_Toc182260541)

[**Supplementary Fig. 2** Calibration plots for observed frequency and predicted probability of prediction model 4](#_Toc182260542)

Supplementary Table 1. Medicine list

| Type of medicine | Medicinal classification | World Health Organization Anatomical Therapeutic Chemical (WHO–ATC) codes |
| --- | --- | --- |
| Antihypertensive agents | Calcium–channel blockers | C08CA |
|  | Angiotensin receptor blockers | C09CA, C09DA, C09DB, C09DX |
|  | Angiotensin–converting enzyme inhibitors | C09AA |
|  | Diuretics | C03AA, C03AX, C03BA, C03CA, C03DB |
|  | Mineralocorticoid receptor blockers | C03DA |
|  | β blockers | C07AA, C07AB, C07AG |
|  | α blockers | C02CA |
| Antidiabetic agents | Insulin | A10A |
|  | Biguanide | A10BA |
|  | Sulfonylurea | A10BB |
|  | Combinations of oral blood glucose lowering drugs | A10BD |
|  | α–glucosidase inhibitors | A10BF |
|  | Thiazolidine | A10BG |
|  | DPP4–i | A10BH |
|  | GLP–1 receptor agonist | A10BJ |
|  | SGLT2–i | A10BK |
|  | Glinide | A10BX |
| NSAIDs |  | M01A |
| Potassium binders | Polystyrene sulfonate | V03AE01 |
|  | Sodium zirconium cyclosilicate | V03AE10 |

DPP4–i, Dipeptidyl peptidase-4 inhibitors; GLP–1, Glucagon-like peptide-1; SGLT2–i, Sodium glucose cotransporter inhibitors; NSAIDs, non-steroidal anti-inflammatory drugs

Supplementary Table 2. Disease names and their International Classification of Diseases 10th Revision (ICD–10) codes

| ICD–10 code | Disease name |
| --- | --- |
|  |  |
| N18.0 | End–stage kidney disease |
| N18.5 | Chronic kidney disease, stage 5 |
| Z94.0 | Kidney transplant status |
| T85.7 | Infection and inflammatory reaction due to other internal prosthetic devices, implants and grafts |

ICD-10, International Classification of Diseases 10th Revision

Supplementary Table 3. Baseline characteristics and modality of kidney replacement therapy

|  | N | Total (N=7,435) | eGFR, mL/min/1.73 m^2^ | | | *P* value |
| --- | --- | --- | --- | --- | --- | --- |
|  |  |  | **>60 (N=1,253)** | **15–60 (N=5,965)** | **15< (N=217)** |  |
| Age, years | 7,435 | 69.1 (11.7) | 65.7 (12.4) | 70.0 (11.3) | 64.4 (13.6) | <0.001 |
| Male sex | 7,435 | 4,262 (57) | 617 (49) | 3,495 (59) | 150 (69) | <0.001 |
| White blood cell, ×10^6^/μl | 7,191 | 6.11 (2.79) | 6.10 (2.47) | 6.11 (2.86) | 6.31 (2.41) | 0.57 |
| Red blood cell, ×10^6^/μl | 7,191 | 4.25 (0.62) | 4.23 (0.63) | 4.28 (0.61) | 3.76 (0.63) | <0.001 |
| Hemoglobin, g/dL | 7,191 | 13.0 (1.8) | 12.9 (1.9) | 13.1 (1.8) | 11.3 (1.6) | <0.001 |
| Hematocrit, % | 7,191 | 39 (5) | 39 (5) | 40 (5) | 35 (5) | <0.001 |
| MCH, pg | 7,191 | 31 (2) | 31 (3) | 31 (2) | 30 (2) | 0.020 |
| MCHC, % | 7,191 | 33 (1) | 33 (1) | 33 (1) | 32 (1) | <0.001 |
| MCV, fL | 7,191 | 93 (6) | 93 (6) | 93 (6) | 93 (6) | 0.48 |
| Platelet, ×1000/μl | 7,191 | 208 (74) | 217 (81) | 207 (69) | 205 (135) | <0.001 |
| Total Protein, mg/dL | 7,385 | 6.9 (0.5) | 6.8 (0.6) | 6.9 (0.5) | 6.6 (0.7) | <0.001 |
| Albumin, g/dL | 7,394 | 4.0 (0.4) | 3.9 (0.5) | 4.0 (0.4) | 3.6 (0.5) | <0.001 |
| HbA1c, % | 2,953 | 6.2 (5.8–6.9) | 6.3 (5.7–7.1) | 6.2 (5.8–6.9) | 6.1 (5.5–7.0) | 0.58 |
| ALT, U/L | 7,390 | 21 (88) | 24 (40) | 21 (96) | 13 (10) | 0.23 |
| AST, U/L | 7,389 | 25 (144) | 26 (33) | 26 (160) | 16 (8) | 0.60 |
| ALP, U/L | 7,285 | 223 (179–278) | 229 (182–291) | 222 (179–275) | 234 (185–321) | <0.001 |
| γ–GTP, U/L | 7,261 | 23 (16–38) | 24 (17–42) | 23 (16–37) | 19 (13–33) | <0.001 |
| LDH, U/L | 7,316 | 190 (168–218) | 192 (167–221) | 189 (168–217) | 198 (171–232) | 0.021 |
| BUN, mg/dL | 7,428 | 20.7 (9.5) | 16.3 (4.3) | 20.5 (7.3) | 52.3 (19.6) | <0.001 |
| eGFR, mL/min/1.73 m^2^ | 7,435 | 52.4 (43.0–58.4) | 64.6 (62.0–69.6) | 50.4 (42.0–55.9) | 6.8 (5.1–10.0) | <0.001 |
| Uric acid, mg/dL | 7,357 | 5.9 (5.0–6.8) | 5.2 (4.4–6.1) | 6.0 (5.1–6.9) | 6.0 (4.8–7.3) | <0.001 |
| Sodium, mEq/L | 7,388 | 141 (140–143) | 141 (140–143) | 141 (140–143) | 140 (137–141) | <0.001 |
| Chloride, mEq/L | 7,382 | 105 (103–107) | 105 (103–106) | 105 (103–107) | 102 (98–105) | <0.001 |
| Na-Cl gap, mEq/L | 7,382 | 36 (35–38) | 37 (35–38) | 36 (35–38) | 37 (35–40) | <0.001 |
| Potassium, mEq/L | 7,396 | 4.4 (0.4) | 4.2 (0.4) | 4.4 (0.4) | 4.6 (0.6) | <0.001 |
| Calcium, mg/dL | 6,666 | 9.2 (8.9–9.5) | 9.2 (8.9–9.4) | 9.2 (9.0–9.5) | 8.8 (8.4–9.2) | <0.001 |
| Phosphorus, mg/dL | 3,694 | 3.3 (2.9–3.7) | 3.2 (2.8–3.7) | 3.3 (2.9–3.7) | 4.5 (3.9–5.3) | <0.001 |
| Magnesium, mg/dL | 3,459 | 2.0 (1.9–2.1) | 2.0 (1.9–2.1) | 2.0 (1.9–2.1) | 2.1 (1.9–2.2) | 0.019 |
| CK, U/L | 5,998 | 91 (64–133) | 84 (59–120) | 93 (65–135) | 96 (66–156) | <0.001 |
| BNP, pg/ml | 876 | 69 (29–187) | 58 (24–174) | 69 (29–185) | 93 (49–260) | 0.039 |
| CRP, mg/dL | 5,715 | 0.08 (0.04–0.24) | 0.09 (0.04–0.32) | 0.08 (0.04–0.22) | 0.12 (0.04–0.43) | <0.001 |
| Cholinesterase, U/L | 2,797 | 290 (244–337) | 286 (230–340) | 294 (249–337) | 240 (201–284) | <0.001 |
| Total cholesterol, mg/dL | 5,248 | 189 (164–215) | 191 (164–221) | 189 (164–214) | 172 (147–203) | <0.001 |
| HDL cholesterol, mg/dL | 4,063 | 52 (42–64) | 54 (44–66) | 52 (42–64) | 46 (39–58) | <0.001 |
| LDL cholesterol, mg/dL | 4,186 | 104 (84–125) | 107 (86–129) | 104 (84–124) | 90 (72–116) | <0.001 |
| Triglyceride, mg/dL | 4,528 | 118 (84–172) | 114 (79–168) | 119 (85–172) | 121 (87–176) | 0.065 |
| Total bilirubin, mg/dL | 6,716 | 0.7 (0.5–0.9) | 0.7 (0.5–0.9) | 0.7 (0.6–0.9) | 0.5 (0.4–0.6) | <0.001 |
| Disease code (ICD 10 code) | | | | | | |
| N18.0 | 7,435 | 54 (1) | 2 (0) | 26 (0) | 26 (12) | <0.001 |
| N18.5 | 7,435 | 4 (0) | 0 (0) | 2 (0) | 2 (1) | <0.001 |
| Z94.0 | 7,435 | 99 (1) | 8 (1) | 86 (1) | 5 (2) | 0.035 |
| T85.7 | 7,435 | 60 (1) | 0 (0) | 10 (0) | 50 (23) | <0.001 |
| Prescription medicine | | | | | | |
| RAS inhibitors | 7,435 | 1,586 (21) | 258 (21) | 1,247 (21) | 81 (37) | <0.001 |
| α blockers | 7,435 | 123 (2) | 18 (1) | 79 (1) | 26 (12) | <0.001 |
| β blockers | 7,435 | 754 (10) | 103 (8) | 599 (10) | 52 (24) | <0.001 |
| CCB | 7,435 | 1,411 (19) | 225 (18) | 1,091 (18) | 95 (44) | <0.001 |
| Diuretics | 7,435 | 757 (10) | 120 (10) | 563 (9) | 74 (34) | <0.001 |
| MRB | 7,435 | 371 (5) | 61 (5) | 253 (4) | 57 (26) | <0.001 |
| Anti diabetics | 7,435 | 1,089 (15) | 215 (17) | 829 (14) | 45 (21) | <0.001 |
| NSAIDs | 7,435 | 511 (7) | 173 (14) | 329 (6) | 9 (4) | <0.001 |
| Potassium binders | 7,435 | 91 (1) | 7 (1) | 63 (1) | 21 (10) | <0.001 |
| PD solutions | 7,435 | 74 (1) | 0 (0) | 8 (0) | 66 (30) | <0.001 |
| Clinical setting | 7,435 |  |  |  |  | <0.001 |
| Outpatient |  | 6936 (93) | 5794 (86) | 5660 (95) | 194 (89) |  |
| Hospitalized |  | 499 (7) | 171 (14) | 305 (5) | 23 (11) |  |
| Modality of KRT at baseline | | | | | | |
| HD | 7,435 | 69 (0.93%) | 0 (0%) | 3 (0.05%) | 66 (30.4%) | <0.001 |
| PD | 7,435 | 48 (0.65%) | 0 (0%) | 0 (0%) | 48 (22.1%) | <0.001 |
| Transplantation | 7,435 | 94 (1.26%) | 9 (0.72%) | 85 (1.42%) | 0 (%) | <0.001 |

Data are expressed as the mean (SD) or median (IQR) for continuous variables and n (%) for categorical variables. Statistical significance was set at P < 0.05. WBC, White blood cell; RBC, Red blood cell; MCH, Mean corpuscular hemoglobin; MCHC, Mean corpuscular hemoglobin concentration; MCV, Mean corpuscular volume; TP, Total protein; AST, Aspartate aminotransferase; ALT, Alanine aminotransferase; ALP, Alkaline phosphatase; JSCC, Japan Society of Clinical Chemistry; γ-GTP, Gamma-glutamyl transpeptidase; LDH, Lactate dehydrogenase; BUN, Blood urea nitrogen; eGFR, Estimated glomerular filtration rate; Mg, Magnesium; CK, Creatine kinase; BNP, Brain natriuretic peptide; CRP, C-reactive protein; HDL, High-density lipoprotein; LDL, Low-density lipoprotein; RAS, Renin-angiotensin system; CCB, Calcium-channel blockers; MRB, Mineralocorticoid receptor blockers; NSAIDs, Non-steroidal anti-inflammatory drugs; KRT, kidney replacement therapy; HD, hemodialysis; PD, peritoneal dialysis

Supplementary Table 4. Logistic regression analysis to develop a classification model

|  | Obs | Univariate | | Multivariable model (adjusted for age, sex, and eGFR) | |
| --- | --- | --- | --- | --- | --- |
|  |  | OR (95% CI) | P value | OR (95% CI) | P value |
| WBC, per 10^6^/µL | 726 | 0.96 (0.90–1.02) | 0.19 | 0.95 (0.88–1.03) | 0.24 |
| RBC, per 10^6^/µL | 726 | 1.66 (1.28–2.15) | <0.001 | 1.61 (1.16–2.24) | 0.004 |
| Hemoglobin, per 1 g/dL | 726 | 1.33 (1.21–1.48) | <0.001 | 1.34 (1.18–1.53) | <0.001 |
| Hematocrit, per 1% | 726 | 1.11 (1.07–1.14) | <0.001 | 1.12 (1.07–1.17) | <0.001 |
| MCH, per 1 pg | 720 | 1.09 (1.03–1.16) | 0.006 | 1.12 (1.03–1.21) | 0.006 |
| MCHC, per 1% | 718 | 0.86 (0.75–0.97) | 0.016 | 0.75 (0.62–0.89) | 0.001 |
| MCV, per 1 fL | 721 | 1.05 (1.03–1.08) | <0.001 | 1.08 (1.04–1.11) | <0.001 |
| Platelet, per 10^4^/µL | 726 | 1.01 (1.00–1.03) | 0.079 | 1.02 (1.00–1.04) | 0.069 |
| TP, per 1 g/dL | 469 | 0.77 (0.58–1.01) | 0.055 | 0.96 (0.69–1.34) | 0.82 |
| Albumin, per 1 g/dL | 701 | 0.58 (0.43–0.80) | 0.001 | 0.67 (0.45–0.98) | 0.041 |
| HbA1c, per 1% | 317 | 0.94 (0.77–1.16) | 0.58 | 0.87 (0.67–1.14) | 0.32 |
| AST* | 676 | 0.51 (0.36–0.72) | <0.001 | 0.98 (0.63–1.52) | 0.92 |
| ALT* | 702 | 0.72 (0.55–0.93) | 0.013 | 0.77 (0.55–1.10) | 0.15 |
| ALP (JSCC)* | 621 | 1.74 (1.17–2.60) | 0.007 | 2.63 (1.56–4.46) | <0.001 |
| γ-GTP* | 653 | 1.03 (0.84–1.26) | 0.79 | 1.42 (1.09–1.85) | 0.009 |
| LDH (JSCC)* | 624 | 0.66 (0.36–1.21) | 0.18 | 0.55 (0.25–1.22) | 0.14 |
| BUN, per 1 mg/dL | 724 | 0.95 (0.95–0.96) | <0.001 | 0.92 (0.90–0.93) | <0.001 |
| eGFR, per 1 mL/min/1.73 m^2^ | 763 | 0.59 (0.55–0.64) | <0.001 | 0.59 (0.55–0.64) | <0.001 |
| Uric acid, per 1 mg/dL | 657 | 0.64 (0.58–0.72) | <0.001 | 0.56 (0.49–0.64) | <0.001 |
| Sodium, per 1 mEq/L | 678 | 0.95 (0.90–0.99) | 0.029 | 1.04 (0.98–1.11) | 0.19 |
| Chloride, per 1 mEq/L | 645 | 0.79 (0.76–0.83) | <0.001 | 0.89 (0.85–0.94) | <0.001 |
| Na – Cl, per 1 mEq/L | 645 | 1.45 (1.36–1.54) | <0.001 | 1.28 (1.20–1.37) | <0.001 |
| Potassium, per 1 mEq/L | 721 | 0.76 (0.61–0.95) | 0.015 | 0.53 (0.39–0.72) | <0.001 |
| Calcium, per 1 mg/dL | 628 | 1.17 (0.93–1.48) | 0.18 | 1.33 (0.99–1.79) | 0.059 |
| Phosphorus, per 1 mg/dL | 540 | 1.41 (1.19–1.66) | <0.001 | 0.64 (0.51–0.79) | <0.001 |
| Mg, per 1 mg/dL | 304 | 3.18 (1.50–6.71) | 0.002 | 2.24 (0.94–5.32) | 0.068 |
| CK* | 475 | 0.43 (0.32–0.56) | <0.001 | 0.25 (0.17–0.38) | <0.001 |
| BNP* | 259 | 1.17 (0.97–1.41) | 0.11 | 1.30 (1.00–1.70) | 0.054 |
| CRP*, per 1 mg/dL | 603 | 1.10 (1.00–1.21) | 0.062 | 1.18 (1.04–1.33) | 0.012 |
| Cholinesterase, per 1 U/L | 270 | 0.999 (0.996–1.002) | 0.62 | 0.998 (0.994–1.002) | 0.33 |
| Total cholesterol, per 1 mg/dL | 445 | 0.998 (0.993–1.002) | 0.37 | 1.003 (0.997–1.01) | 0.36 |
| HDL cholesterol, per 1 mg/dL | 413 | 1.001 (0.992–1.011) | 0.79 | 1.017 (1.003–1.031) | 0.02 |
| LDL cholesterol, per 1 mg/dL | 376 | 1.004 (0.997–1.01) | 0.26 | 1.006 (0.996–1.015) | 0.24 |
| Triglyceride, per 1 mg/dL | 475 | 0.998 (0.996–1.00) | 0.076 | 0.998 (0.995–1.00) | 0.091 |
| Total bililubin, per 1 mg/dL | 543 | 0.81 (0.54–1.22) | 0.31 | 1.25 (0.87–1.78) | 0.23 |
| N18.0 | 763 | 5.16 (3.66–7.28) | <0.001 | 3.70 (2.44–5.61) | <0.001 |
| N18.5 | 763 | 0.89 (0.53–1.50) | 0.67 | 1.05 (0.54–2.04) | 0.90 |
| T85.7 | 763 | 16.1 (5.81–44.6) | <0.001 | 3.76 (1.29–11.0) | 0.016 |
| RAS inhibitors | 763 | 0.24 (0.17–0.33) | <0.001 | 0.21 (0.14–0.33) | <0.001 |
| α blockers | 763 | 0.43 (0.27–0.71) | 0.001 | 0.38 (0.20–0.72) | 0.003 |
| β blockers | 763 | 0.59 (0.39–0.88) | 0.010 | 0.38 (0.22–0.64) | <0.001 |
| CCB | 763 | 0.23 (0.16–0.31) | <0.001 | 0.18 (0.11–0.28) | <0.001 |
| Diuretics | 763 | 0.48 (0.34–0.68) | <0.001 | 0.40 (0.25–0.63) | <0.001 |
| MRB | 763 | 1.69 (1.01–2.85) | 0.047 | 0.81 (0.40–1.66) | 0.57 |
| Anti diabetics | 763 | 0.77 (0.54–1.10) | 0.16 | 0.84 (0.53–1.32) | 0.44 |
| NSAIDs | 763 | 0.89 (0.34–2.33) | 0.81 | 0.54 (0.18–1.63) | 0.28 |
| Potassium binders | 763 | 0.11 (0.06–0.21) | <0.001 | 0.13 (0.06–0.27) | <0.001 |

Multivariable model was adjusted for age, sex, and eGFR.

*Logarithmic transformations were performed and included in the models.

WBC, White blood cell; RBC, Red blood cell; MCH, Mean corpuscular hemoglobin; MCHC, Mean corpuscular hemoglobin concentration; MCV, Mean corpuscular volume; TP, Total protein; AST, Aspartate aminotransferase; ALT, Alanine aminotransferase; ALP, Alkaline phosphatase; JSCC, Japan Society of Clinical Chemistry; γ-GTP, Gamma-glutamyl transpeptidase; LDH, Lactate dehydrogenase; BUN, Blood urea nitrogen; eGFR, Estimated glomerular filtration rate; Mg, Magnesium; CK, Creatine kinase; BNP, Brain natriuretic peptide; CRP, C-reactive protein; HDL, High–density lipoprotein; LDL, Low-density lipoprotein; RAS, Renin-angiotensin system; CCB, Calcium–channel blockers; MRB, Mineralocorticoid receptor blockers; NSAIDs, Non-steroidal anti-inflammatory drugs.

Supplementary Table 5. The model performance at each hospital

|  | HD or PD | Not on dialysis | Sensitivity | Specificity | PPV | NPV |
| --- | --- | --- | --- | --- | --- | --- |
| **Hospital 1** | | | | | | |
| **≥Cut point** | 104 | 24 | 93.7% | 76.2% | 81.2% | 91.7% |
| **<Cut point** | 7 | 77 |  |  |  |  |
| **Hospital 2** | | | | | | |
| **≥Cut point** | 53 | 6 | 86.9% | 91.2% | 89.8% | 88.6% |
| **<Cut point** | 8 | 62 |  |  |  |  |
| **Hospital 3** | | | | | | |
| **≥Cut point** | 79 | 3 | 95.2% | 94.9% | 96.3% | 93.3% |
| **<Cut point** | 4 | 56 |  |  |  |  |
| **Hospital 4** | | | | | | |
| **≥Cut point** | 162 | 7 | 94.7% | 93.6% | 95.9% | 91.9% |
| **<Cut point** | 9 | 102 |  |  |  |  |
| **Hospital 5** | | | | | | |
| **≥Cut point** | 62 | 2 | 91.2% | 96.8 | 96.9% | 90.9% |
| **<Cut point** | 6 | 60 |  |  |  |  |
| **Hospital 6** | | | | | | |
| **≥Cut point** | 143 | 6 | 97.9% | 94.2% | 96.0% | 97% |
| **<Cut point** | 3 | 97 |  |  |  |  |

HD, Hemodialysis; PD, Peritoneal dialysis; PPV, Positive predictive value; NPV, Negative predictive value

Supplementary Table 6. Performance of other Models

|  | HD or PD | Not on dialysis | Sensitivity | Specificity | PPV | NPV |
| --- | --- | --- | --- | --- | --- | --- |
| **Model 2:** eGFR, BUN, UA, Na–Cl gap, and Alb | | | | | | |
| **Derivation cohort**: AUC = 0.9487 (95% confidence interval: 0.9315–0.9658) | | | | | | |
| ≥Cut point | 395 | 41 | 92.7% | 87.8% | 90.6% | 90.5% |
| <Cut point | 31 | 296 |  |  |  |  |
| **Validation cohort**: AUC = 0.9755 (95% confidence interval: 0.9580–0.9929) | | | | | | |
| ≥Cut point | 202 | 5 | 94.4% | 97.0% | 97.6% | 93.0% |
| <Cut point | 12 | 160 |  |  |  |  |
| **Model 3:** Model 2 + RBC and hematocrit | | | | | | |
| **Derivation cohort**: AUC = 0.9500 (95% confidence interval: 0.9331–0.9669) | | | | | | |
| ≥Cut point | 389 | 35 | 91.3% | 89.6% | 91.7% | 89.1% |
| <Cut point | 37 | 302 |  |  |  |  |
| **Validation cohort**: AUC = 0.9772 (95% confidence interval: 0.9605–0.9939) | | | | | | |
| ≥Cut point | 200 | 5 | 93.5% | 97.0% | 97.6% | 92.0% |
| <Cut point | 14 | 160 |  |  |  |  |
| **Model 4:** Model 1 + N18.0, T85.7, and use of calcium-channel blockers | | | | | | |
| **Derivation cohort**: AUC = 0.9593 (95% confidence interval: 0.9449–0.9737) | | | | | | |
| ≥Cut point | 397 | 31 | 93.2% | 90.8% | 92.8% | 91.3% |
| <Cut point | 29 | 306 |  |  |  |  |
| **Validation cohort**: AUC = 0.9787 (95% confidence interval: 0.9630–0.9944) | | | | | | |
| ≥Cut point | 194 | 5 | 90.7% | 97.0% | 97.5% | 88.9% |
| <Cut point | 20 | 160 |  |  |  |  |

HD, Hemodialysis; PD, Peritoneal dialysis; PPV, Positive predictive value; NPV, Negative predictive value

Supplementary Table 7. Performance of classification models after excluding hospitalized patients

|  | **HD or PD** | **Not on dialysis** | **Sensitivity** | **Specificity** | **PPV** | **NPV** |
| --- | --- | --- | --- | --- | --- | --- |
| **Model 1:** eGFR, BUN, UA, Na–Cl gap, Alb, MCH, and MCHC | | | | | | |
| **Derivation cohort**: AUC = 0.9536 (95% confidence interval: 0.9357–0.9715) | | | | | | |
| ≥Cut point | 344 | 31 | 94.2% | 89.7% | 91.7% | 92.8% |
| <Cut point | 21 | 269 |  |  |  |  |
| **Validation cohort**: AUC = 0.9755 (95% confidence interval: 0.9580–0.9929) | | | | | | |
| ≥Cut point | 177 | 8 | 95.2% | 94.5% | 95.%7 | 93.8% |
| <Cut point | 9 | 137 |  |  |  |  |
| **Model 2:** eGFR, BUN, UA, Na–Cl gap, and Alb | | | | | | |
| **Derivation cohort**: AUC = 0.9522 (95% confidence interval: 0.9338–0.9707) | | | | | | |
| ≥Cut point | 342 | 31 | 93.7% | 89.7% | 91.7% | 92.1% |
| <Cut point | 23 | 269 |  |  |  |  |
| **Validation cohort**: AUC = 0.9723 (95% confidence interval: 0.952405–0.9922) | | | | | | |
| ≥Cut point | 175 | 5 | 94.1% | 96.6% | 97.2% | 92.7% |
| <Cut point | 11 | 140 |  |  |  |  |
| **Model 3:** Model 2 + RBC and hematocrit | | | | | | |
| **Derivation cohort**: AUC = 0.9593 (95% confidence interval: 0.9449–0.9737) | | | | | | |
| ≥Cut point | 337 | 26 | 92.3% | 91.3% | 92.8% | 90.7% |
| <Cut point | 28 | 274 |  |  |  |  |
| **Validation cohort**: AUC = 0.9787 (95% confidence interval: 0.9630–0.9944) | | | | | | |
| ≥Cut point | 173 | 5 | 93.0% | 96.6% | 97.2% | 91.5% |
| <Cut point | 13 | 140 |  |  |  |  |
| **Model 4:** Model 1 + N18.0, T85.7, and use of calcium-channel blockers | | | | | | |
| **Derivation cohort**: AUC = 0.9593 (95% confidence interval: 0.9449–0.9737) | | | | | | |
| ≥Cut point | 345 | 27 | 94.5% | 91.0% | 92.7% | 93.2% |
| <Cut point | 20 | 273 |  |  |  |  |
| **Validation cohort**: AUC = 0.9787 (95% confidence interval: 0.9630–0.9944) | | | | | | |
| ≥Cut point | 167 | 6 | 89.8% | 95.9% | 96.5% | 88.0% |
| <Cut point | 19 | 139 |  |  |  |  |

After excluding hospitalized patients, the derivation cohort contains 665 patients and the validation cohort contains 331 patients. HD, Hemodialysis; PD, Peritoneal dialysis; PPV, Positive predictive value; NPV, Negative predictive value


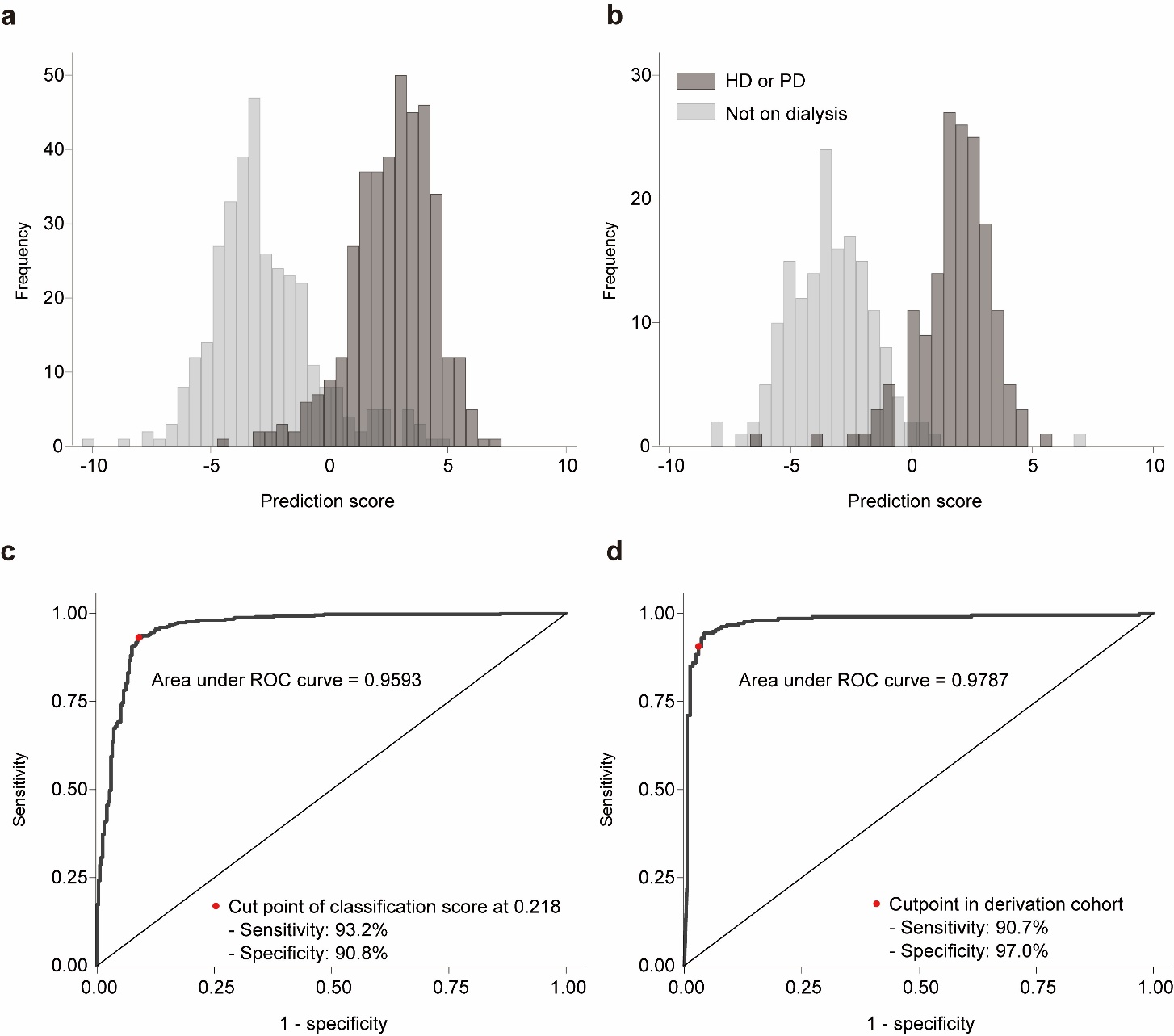


Supplementary Fig. 1 Distribution of prediction scores 4 and receiver operating characteristic curves in the derivation and validation cohorts

Distributions of the prediction score in the derivation and validation cohorts (**A** and **B**, respectively). The scores were calculated using the following equation: Prediction score 4 = 0.1688315× MCH – 0.3714643× MCHC –0.8296468× Alb – 0.0752203× BUN – 0.5031476× eGFR + 0.1839276× Na–Cl gap – 0.2339113× UA + 1.357285× T85.7 + 1.467646× N18.0 – 0.7974775× CCB + 12.27361.

The receiver operating characteristic curves show excellent discrimination performance of the model at the cut point of 0.218 in both derivation and validation cohorts (**C** and **D**, respectively).


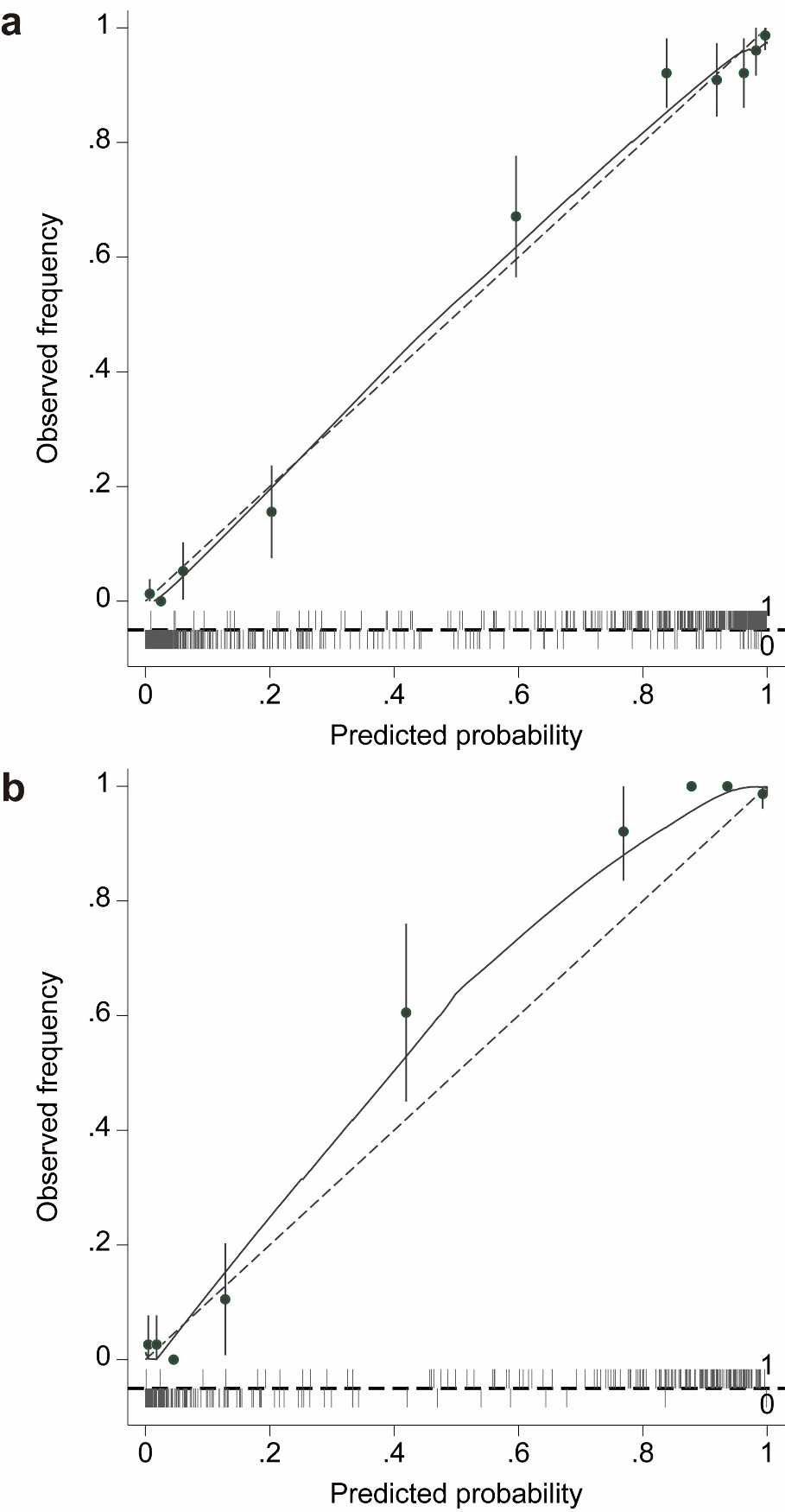


Supplementary Fig. 2 Calibration plots for observed frequency and predicted probability of prediction model 4

**A**. Derivation cohort. **B**. Validation cohort.
